# Role of Dual-Energy Computed Tomography in Characterization of Ureteric Calculi and Urinary Obstruction

**DOI:** 10.7759/cureus.8002

**Published:** 2020-05-07

**Authors:** Anchal Singh, Sachin Khanduri, Nazia Khan, Poonam Yadav, Mushahid Husain, Ahmad Umar Khan, Mazhar Khan, Shreshtha Jain

**Affiliations:** 1 Radiodiagnosis, Era's Lucknow Medical College and Hospital, Lucknow, IND; 2 Radiology, Era's Lucknow Medical College and Hospital, Lucknow, IND; 3 Radiology, Era’s Lucknow Medical College and Hospital, Lucknow, IND; 4 Radiology, All India Institute of Medical Science, Patna, IND

**Keywords:** chemical composition, dual energy ct, obstruction, ureteric calculi

## Abstract

Objective

The present study was carried out to assess the accuracy of dual-energy computed tomography (DECT) in the morphological and chemical characterization of ureteric calculi along with the prediction of the grade of urinary obstruction.

Methods

This was a prospective observational study that included 100 cases with ultrasonography (USG)-diagnosed ureteric calculi that underwent surgery or had spontaneous expulsion of ureteric calculi. At enrolment, DECT was performed for an in vivo evaluation of volume, chemical composition, and grade of obstruction by subjective assessment of the perinephric edema. After surgical intervention, in vitro evaluation of volume was done by fluid displacement followed by infrared spectroscopy (IRS) for chemical composition. DECT findings were compared with the biochemical analysis and degree of obstruction was validated against excretory CT urograms. Sensitivity, specificity, and the positive predictive and negative predictive values of DECT were assessed.

Results

No significant difference was observed between the mean volume of stones by fluid displacement (65.1±77.61 mm^3^) and DECT assessment (66.09±81.78 mm^3^). IRS revealed the composition of stones as hydroxyapatite, uric acid, cysteine, oxalic acid, and mixed type in 48, 23, 15, five, and nine cases. The sensitivity and specificity of DECT for hydroxyapatite, uric acid, cysteine, oxalic acid, and mixed types were 89.6% and 88.5%, 82.6% and 97.5%, 86.7% and 96.5%, 80% and 98.9%, and 88.9% and 98.9%, respectively. On CT urography, a total of 35 had a high-grade and 65 had a low-grade obstruction, whereas DECT revealed high- and low-grade obstructions in 42 and 58 cases. The sensitivity and specificity of DECT for a high-grade obstruction were 94.3% and 86.2%.

Conclusions

The findings of the study showed that DECT provides comprehensive information regarding the morphological, chemical, and anatomical characterization of ureteric stones.

## Introduction

Urolithiasis is a common problem affecting 1%-5% of the population. It has a significant impact on the quality of life and leads to significant morbidity [1-2]. The worldwide prevalence ranges from 5%-9% in Europe, 7%-13% in North America, and 1%-5% in Asia [3].

The characterization of ureteric calculi and an understanding of their chemical composition is helpful from the point of view of their management. The study of urolithiasis is important not only because of the high recurrence rates and incidence but also because of the complications that arise from it like hydronephrosis leading to renal failure in the long run if the stone partially obstructs the urinary tract [4].

Imaging techniques, such as urography and ultrasonography (USG), are helpful in characterizing the morphology, size, number, and, to a certain extent, volume of the ureteric calculi. However, the estimation of the chemical composition of the calculus remains unknown. In recent years, dual-energy computed tomography (DECT) has emerged as a useful method not only for morphological and anatomical evaluation but also for chemical composition [4].

The advantage of DECT has been its ability to provide material-specific information that is unavailable on conventional single-energy CT without a significantly higher radiation dose. DECT is effective for the estimation of volume, character, and degree of obstruction in ureteric calculus [5].

The attenuation patterns and low- and high-energy levels are helpful in spectral separation and thus in understanding the chemical composition of the materials, as lighter materials show small differences in attenuation between low and high energy levels, whereas those heavier materials show larger differences in attenuation between low and high energy levels [6].

Chaytor et al. assessed the accuracy of DECT in characterizing urinary tract stone composition in patients presenting with renal colic and found it useful in the characterization of the renal stones [7]. Ege et al., in another study, assessed the incidence of the secondary signs associated with ureteral stones on unenhanced helical CT of patients with acute renal colic [8]. Out of the 110 patients, 91 had hydroureter, 88 had hydronephrosis, 65 had periureteric edema, and 57.2% had unilateral renal enlargement. A total of 90 stones passed spontaneously and intervention was required in 21 cases. The authors found the CT scans useful in the assessment of the urinary obstruction.

In view of the promising role of DECT in stones characterization and association with urinary obstruction as seen in different studies, the present study was done at our tertiary care center to evaluate the volume of ureteric calculus, determine the composition of calculus materials, and measure the efficacy of DECT in the evaluation of perinephric edema to predict the degree of ureteral obstruction in patients with acute ureterolithiasis.

## Materials and methods

A prospective observations study was done in the Department of Radiodiagnosis over a period of one year. A total of 100 patients with ureteric calculus diagnosed on ultrasound/X-ray kidneys, ureters, and urinary bladder (KUB) in the age group of 18 to 70 years, who underwent invasive procedures, including ureterorenoscopy or who had spontaneous expulsion were enrolled in the study after taking informed consent. Approval from the Institutional Ethics Committee was obtained before starting the study. Pregnant women and the patients allergic to the contrast dye were excluded from the study.

The sample size was estimated based on the study by Ilyas M et al. who observed the sensitivity and specificity of DECT in differentiating a calcium oxalate from non-calcium oxalate calculus was 97.8% and 92.3%, respectively [9]. Taking these values as a reference, the minimum required sample size with the desired precision of 10%, 80% power of study, and 5% level of significance was 72 patients. To reduce the margin of error, the total sample size taken was 100.

The demographic information and presenting complaints of the patients were noted. A thorough history was taken and a physical examination was performed. DECT was performed using Siemens “SOMATOM-force (384 slices)’’ dual-source dual-energy CT machine with tin filter (Siemens AG, Munich, Germany) for an in vivo evaluation of volume and chemical composition. The patient was positioned supine on the CT table with an area of interest being the abdomen. A dual-energy scan was performed, which acquired the image from two tubes angled at 90° - low energy (80 eV) and high energy (140 eV), respectively. The ratio of low- and high-energy attenuation was calculated (the DE ratio). Image acquisition and post-processing image interpretation were done per the technique described by Ilyas et al. [9]. For uric acid, cysteine, mixed, calcium oxalate, and hydroxyapatite calculi, the cut-off values of the DE ratio were taken as <1.13, 1.13-1.23, 1.23-1.33, 1.33-1.53, and >1.53, respectively, and, accordingly, the chemical composition of the calculi was determined [9].

Excretory CT urogram was done to estimate the degree of obstruction as per the protocol of the modified version of the Society for Foetal Urology Hydronephrosis Grading System where no dilatation or local dilatation of the ureter and/or renal pelvis was categorized as low grade and calyceal dilatation along with ureter and renal pelvis dilatation was categorized as high-grade obstruction.

Post-surgical extraction or spontaneous expulsion, the size of the calculi was measured in the largest dimension. As some of the stones were fragmented during the procedure, the volume was assessed using the fluid displacement method (volumetric flask method), which was taken as the gold standard for comparison [10-11]. The chemical composition of the calculi was assessed through infrared spectroscopy (IRS).

DECT was performed on all the patients for an in vivo evaluation of volume and the chemical composition of the stones. The grade of obstruction was predicted on DECT subjectively, after the assessment of the perinephric edema in terms of strands of soft tissue attenuation in perinephric fat, perinephric fluid collection, and thickness of or collection of fluid along the renal fasciae [12-13].

The efficacy of DECT for the determination of chemical composition and the prediction of the grade of obstruction was assessed in terms of sensitivity, specificity, positive predictive value (PPV) and negative predictive value (NPV), and accuracy, respectively.

Statistical analysis

Quantitative data were presented with the help of mean and standard deviation. A comparison among the study groups was done with the help of the unpaired t-test per the results of the normality test. Qualitative data were presented with the help of the frequency and percentage table. Association among the study groups was assessed with the help of the Fisher test, student t-test, and chi-square test. A p-value of less than 0.05 was taken as significant. Appropriate statistical software, including but not restricted to Microsoft Excel (Microsoft Corporation, Redmond, Washington) and SPSS ver. 20 (IBM Corp., Armonk, New York), was used for statistical analysis. Graphical representation was done in Microsoft Excel 2010.

## Results

The age of patients ranged from 18 to 70 years, with a mean age of 45.6±11.7 years. The majority (59%) of patients were males. Eight patients reported a positive family history of urolithiasis. The post-extraction mean calculus size and volume were 9.45±7.13 mm and 65.1±77.61 mm^3^, respectively. The number of stones ranged from one to six, with a mean of 3.8±2.9. Urography revealed a high-grade obstruction in 35% and a low-grade obstruction in 65% cases. Post-extraction IRS revealed the chemical composition of stones as hydroxyapatite in the maximum number of cases (48%) followed by uric acid (23%), cysteine (15%), and calcium oxalate (oxalic acid,5%), respectively (Table 1).

**Table 1:** Patient demographics, general characteristics, and chemical composition of ureteric calculi (n=100) SD: standard deviation

SN	Characteristic	Statistic
1	Mean age ± SD (range) in years	45.6±11.7 (18-70)
2	Gender (n)	
	Male	59
	Female	41
3	Positive family history (n)	8
4	Mean calculus size ± SD (range) in mm	9.45±7.13 (4-22)
5	Mean calculus volume ± SD (range) in mm^3^ (using fluid displacement method)	65.1±77.61 (5.8-271)
6	Mean number of calculi ± SD (range)	3.8±2.9 (1 to 6)
7	Degree of obstruction (excretory urography)	
	High grade	35
	Low grade	65
8	Calculus type as per the chemical analysis	
	Hydroxyapatite	48
	Uric acid	23
	Cysteine	15
	Oxalic acid	5
	Mixed	9

**Figure 1:**
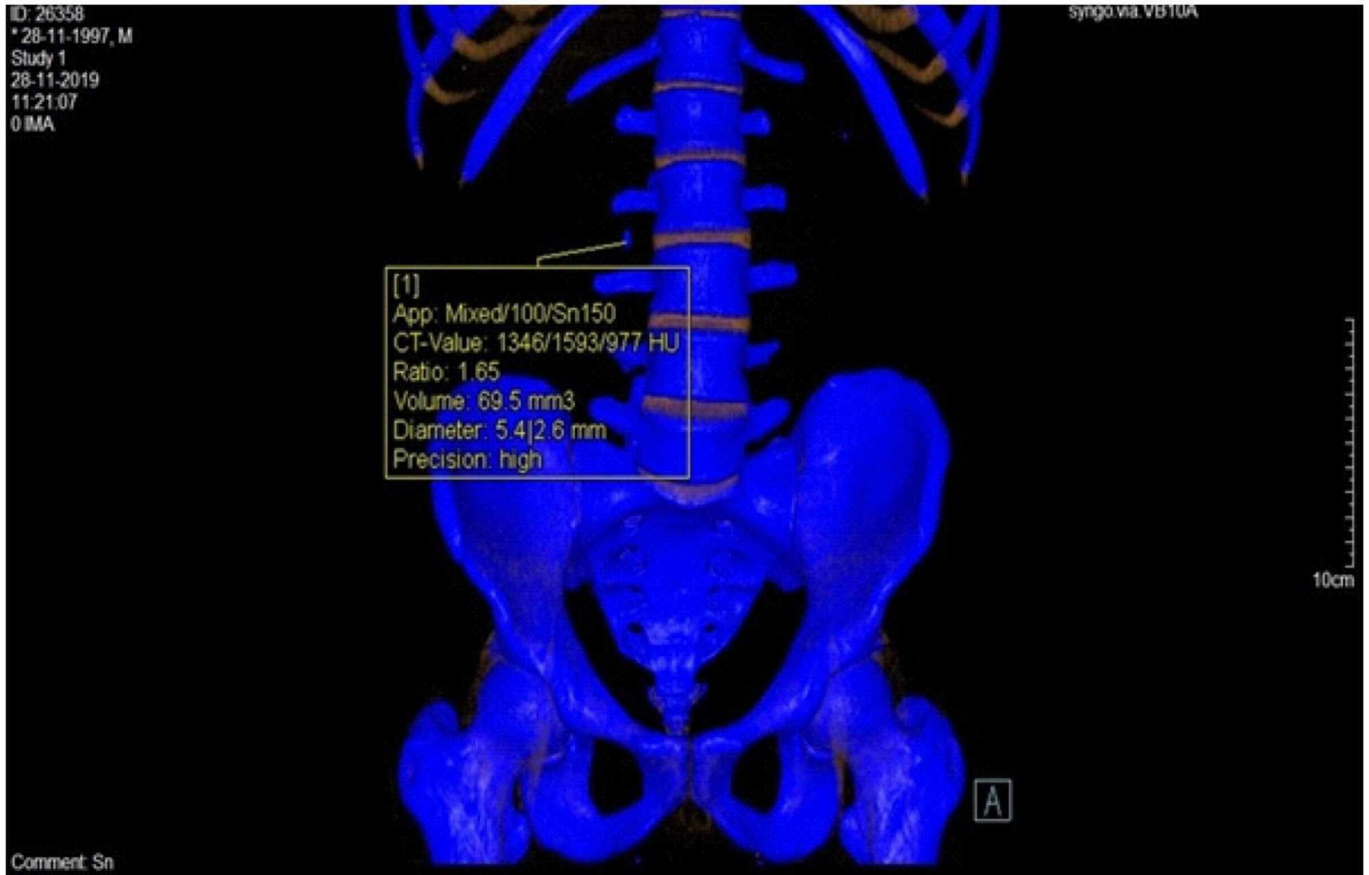
Noncontrast coronal dual-energy CT images with right-sided ureteric calculus CT: computed tomography

**Figure 2:**
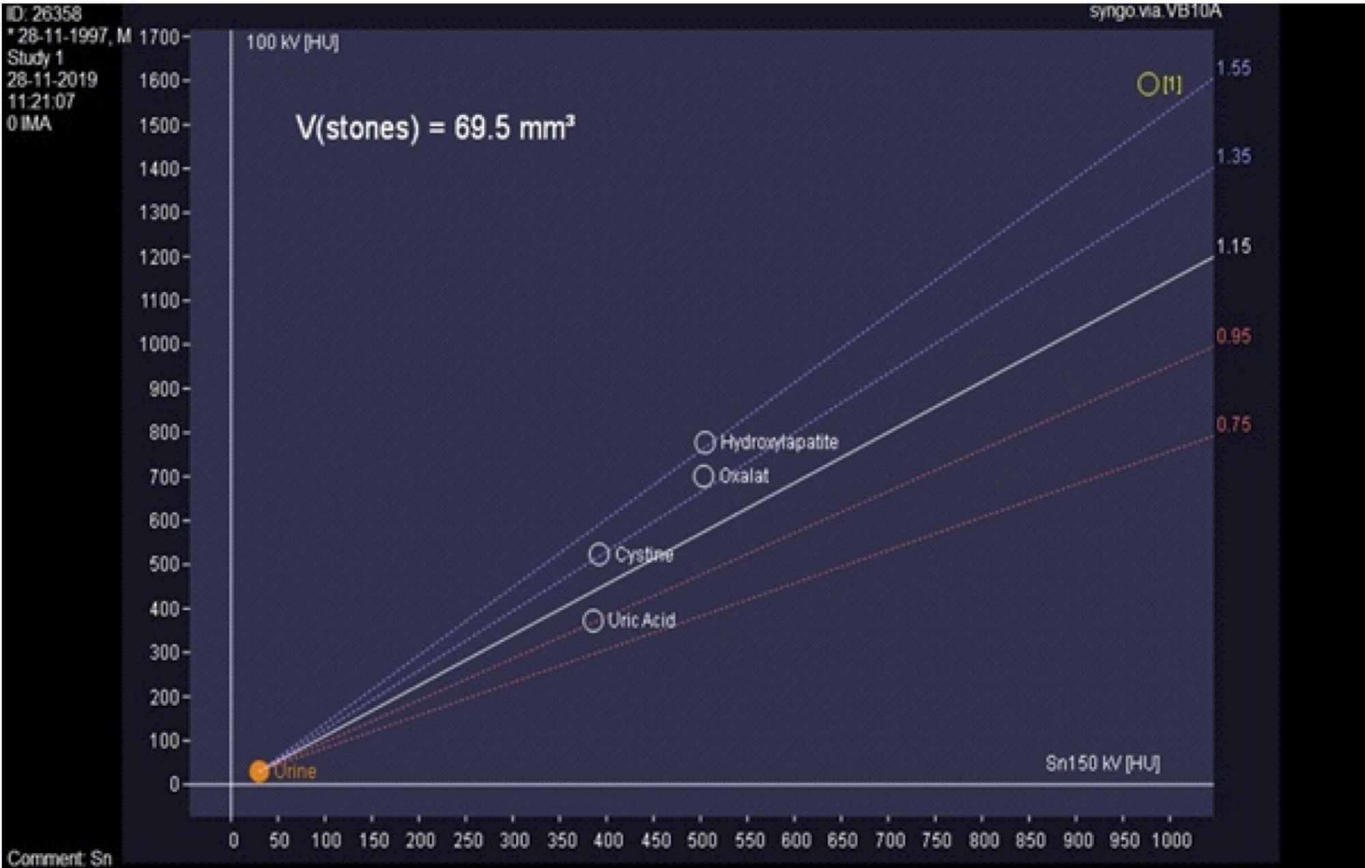
Graph showing the characterization of the right ureteric calculus (labeled 1) as hydroxyapatite stone

**Figure 3:**
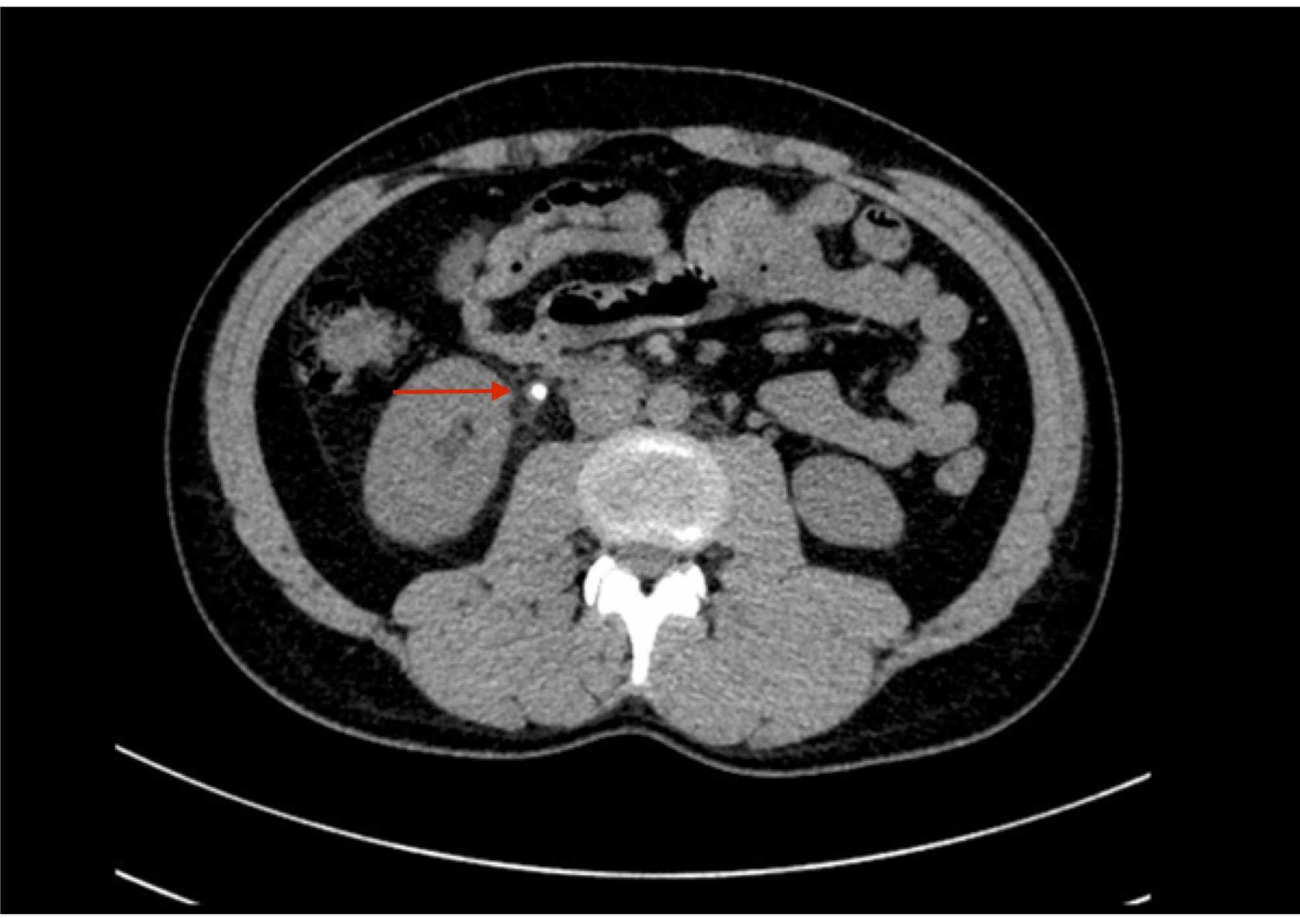
Axial non-contrast DECT image showing a calculus in the right proximal ureter with perinephric and periureteric fat stranding DECT: dual-energy computed tomography

On DECT, the mean CT attenuation values for low energy (80 eV) and high energy (140 eV) were 1271±178 and 758±138 HU, respectively, for hydroxyapatite (mean DE ratio = 1.68), 534±62 and 544±58 HU, respectively, for uric acid (mean DE ratio = 0.98), 1110±146 and 935±168 HU, respectively, for cysteine (mean DE ratio = 1.19) and 853±284 and 569±312 HU, respectively, for oxalic acid (mean DE ratio = 1.50) stones, respectively. For cases with mixed stones, the attenuation values for low-energy and high-energy levels were 1203±327 and 933±289, respectively (mean DE ratio = 1.29). The mean calculus volume as per the DECT measurement was 66.09±81.78 mm^3^. DECT diagnosed 42 cases as high-grade (dilatation of the ureter, renal pelvis, and calyces) and 58 cases as low-grade obstructions (local dilatation of the ureter and/or renal pelvis dilatation). On CT images, subjective assessment of the perinephric edema, fat stranding, and fluid collection suggested high-grade obstruction in 42% cases and low-grade obstruction in 58% cases (Table 2). Figures 1-3 show the DECT image of a right-sided ureteric stone characterized as hydroxyapatite stone along with perinephric and periureteric edema and fat stranding.

**Table 2:** DECT quantitative analysis for different types of calculi, volume, and degree of obstruction DECT: dual-energy computed tomography; HU: Hounsfield unit; SD: standard deviation

Composition	No. of Cases	Mean ± SD (HU) 80 eV	Mean±SD (HU) 140 eV	DE Ratio
Hydroxyapatite	48	1271±178	758±138	1.68
Uric acid	23	534±62	544±58	0.98
Cysteine	15	1110±146	935±168	1.19
Oxalic acid	5	853±284	569±312	1.5
Mixed	9	1203±327	933±289	1.29
Mean calculus volume±SD (range) in mm^3^ on DECT				66.09±81.78 (5.8-271)
High grade				42
Low grade				58

On DECT, a total of 49 calculi were diagnosed as hydroxyapatite, 19 as uric acid, 16 as cysteine, and five as oxalic acid. Nine cases were diagnosed as mixed type on DECT. On comparing the DECT findings with IRS findings, out of 49 cases diagnosed as hydroxyapatite, 43 (87.8%) were true positive while six (12.2%) were false positive (four uric acid and two cysteine calculi on IRS). On the other hand, out of 48 cases diagnosed as hydroxyapatite by IRS, only five (8.3%) were false negative (three diagnosed as cysteine and one each diagnosed as mixed and oxalic acid respectively by DECT), thus 46 out of 52 (88.5%) cases were true negative. Of the 21 cases diagnosed as uric acid by DECT, 19 (90.5%) were true positive while two (9.5%) were false positive (one each diagnosed as oxalic acid and mixed type by IRS). On the other hand, of the 23 cases diagnosed as uric acid by IRS and four (17.4%) were false negative (diagnosed as hydroxyapatite by DECT), thus 75 out of 77 (97.4%) were true negative. Out of 16 cases diagnosed as cysteine calculi by DECT, a total of 13 (81.3%) were true positive while three (18.7%) were false positive (diagnosed as hydroxyapatite by IRS). Of the 15 cases diagnosed as cysteine calculi by IRS, two (13.3%) were false negative (diagnosed as hydroxyapatite by DECT), thus a total of 82 out of 85 (96.5%) were false negative. Both DECT and IRS diagnosed five cases as oxalic acid calculi. There was one false positive (diagnosed as uric acid by IRS) and one false negative (diagnosed as hydroxyapatite by DECT). Both DECT and IRS diagnosed nine cases each as mixed type. There was one false positive (diagnosed as hydroxyapatite by IRS) and one false negative (diagnosed as uric acid by DECT), respectively. Overall, there was an 87% agreement between IRS and DECT. On correlating the DECT-predicted chemical composition with IRS diagnosed chemical composition, the sensitivity, specificity, PPV, NPV, and accuracy of DECT for hydroxyapatite, uric acid, cysteine, oxalic acid, and mixed types were 89.6%, 88.5%, 87.8%, 92.2% and 89%; 82.6%, 97.5%, 90.5%, 94.9% and 94%; 86.7%, 96.5%, 81.3%, 97.6% and 95%; 80%, 98.9%, 80%, 98.9% and 98%; and 88.9%, 98.9%, 88.9%, 98.9% and 98%, respectively (Table 3).

**Table 3:** Correlation between chemical composition and DECT-detected composition of ureteric calculi DECT: dual-energy computed tomography; IR: infrared; NPV: negative predictive value; PPV: positive predictive value

DECT-Detected Composition	Chemical Composition (IR Spectroscopy)					
	Hydroxyapatite	Uric acid	Cysteine	Oxalic acid	Mixed	Total
Hydroxyapatite	43	4	2	0	0	49
Uric acid	0	19	0	1	1	21
Cysteine	3	0	13	0	0	16
Oxalic acid	1	0	0	4	0	5
Mixed	1	0	0	0	8	9
Total	48	23	15	5	9	100
Chemical Composition	Diagnostic Efficacy of DECT					
	Sensitivity	Specificity	PPV	NPV	Accuracy	
Hydroxyapatite	89.60%	88.50%	87.80%	92.20%	89.00%	
Uric acid	82.60%	97.50%	90.50%	94.90%	94.00%	
Cysteine	86.70%	96.50%	81.30%	97.60%	95.00%	
Oxalic acid	80.00%	98.90%	80.00%	98.90%	98.00%	
Mixed	88.90%	98.90%	88.90%	98.90%	98.00%	

Of the 35 cases diagnosed as high-grade obstruction, 33 (94.3%) were true positive on DECT while 2 (5.7%) were false negative whereas of the 65 cases diagnosed as low grade (not high grade) on urogram, 56 (86.2%) were true negative and 9 (13.8%) were false positive. Correspondingly, the sensitivity, specificity, PPV, NPV, and accuracy of DECT for the detection of urography-diagnosed high-grade obstruction was 94.3%, 86.2%, 78.6%, 96.6%, and 89% respectively (Table 4).

**Table 4:** Correlation between the degree of obstruction by urogram and DECT DECT: dual-energy computed tomography; NPV: negative predictive value; PPV: positive predictive value

DECT Analysis	Urogram			
	High grade	Low grade	Total	
High grade	33	9	42	
Low grade	2	56	58	
Total	35	65	100	
Sensitivity	Specificity	PPV	NPV	Accuracy
94.30%	86.20%	78.60%	96.60%	89%

## Discussion

The management of the ureteric stones depends upon the anatomical, morphological, and chemical characterization of the stones. It becomes imperative to know the features of the stone before operating, so as to provide the best management to the patient.

The present study found a high accuracy of DECT in the morphological assessment (volume of calculus) as well as the chemical composition. The mean calculus volume as per fluid displacement was 65.1±77.61 mm^3^, which did not differ significantly as compared to the DECT-measured calculus volume, which was 66.09±81.78 mm^3^. The mean difference between the DECT and in vitro assessment was <1 mm^3^, showing high accuracy. The management of stone volume has a detrimental role in the choice of therapeutic approach. As per Narepalem et al., volume differences >4.1 mm^3^ are clinically significant from the point of view of the selection of treatment options, however, DECT findings in the present study showed that the volume differences were not significant [11]. Similar observations were made by some previous studies too [12-14].

With respect to the degree of obstruction, the present study showed that DECT was 94.3% sensitive and 86.2% specific for the detection of high-grade obstructions as observed on excretory urogram. However, the accuracy of DECT was only 89%, the reason for this could be a high false-positive rate (21.4%) due to a subjective assessment of the degree of perinephric edema. However, one must understand that an excretory urogram is another imaging technique that has its own limitations in assessing the degree of obstruction and thus the correlations among both of them may further add to the false-positive rates. As compared to the index study, Boridy et al. in their study had shown the CT evaluation for the degree of obstruction was 100% sensitive and 100% specific in view of intraoperative correlation [12]. These findings, in turn, suggest an additive role of DECT in the evaluation of the degree of obstruction.

As far as the chemical composition was concerned, we observed an 87% agreement between DECT and IRS. Among different types of calculi, maximum sensitivity was observed for hydroxyapatite (89.6%) and mixed types (88.9%) while maximum specificity was observed for mixed type and oxalic acid (98.9% each). In fact, hydroxyapatite was the dominant type in the present study, seen in as many as 48% of cases by IRS and 49% of cases by DECT. This was followed by uric acid (23%), cysteine (15%), and oxalic acid (5%) calculi while mixed stones were detected in 9% of cases. Compared to the present study, Erdogan et al. in their study found oxalic acid (42.9%) and hydroxyapatite (36.5%) as the dominant types on DECT while the in vitro analysis showed 22.9% as hydroxyapatite and 51.4% as oxalic acid calculi [15]. In another study, Ilyas et al. found oxalic acid as the dominant type seen in 78.4% of their cases [9]. Manglaviti et al. in their study did not diagnose hydroxyapatite in any of the cases and reported a dominance of oxalic acid (67.3%) calculi [16]. In another study, Basha et al. described the chemical composition of ureteric calculi as calcium oxalate or phosphate (75.7%) followed by uric acid (27.0%) and cysteine stones (10.8%), respectively [17]. In the present study, the proportion of hydroxyapatite and oxalic acid stones together was 53% followed by uric acid (23%) and cysteine (15%), respectively. As far as differentiation between uric acid and non-uric acid stones is concerned, the present study is in concordance with the observations of Ilyas et al. who found 85% of their sample to be non-uric acid and only 15% to be uric acid stones [9]. The reason for the difference in the chemical composition of stones in different studies could be attributable to the inclusion criteria used and the difference in dietary habits and ethnicity of the affected population. In addition, the different DE ratio cutoffs used for the characterization of ureteric stones among different studies may cause the difference in the results, necessitating the standardization of the DE ratios for in vivo stone characterization.

The high accuracy of DECT in the identification of the chemical composition of ureteric calculi, as observed in the present study (87%), is in agreement with the observation made by Erdogan et al. who reported the accuracy of DECT to be 91.4% [15]. Manglaviti et al. in their study also reported an agreement in 45/49 (91.8%) cases [16]. In the present study, the highest accuracy of stone detection was observed for oxalic acid and mixed types (98%), whereas for hydroxyapatite, though the sensitivity was the maximum (89.6%), the accuracy was minimum (89%). This was owing to both high false positive (n=6) as well as false negative (n=5) rates. For uric acid and cysteine stones, the accuracy was 94% and 95%, respectively. Ilyas et al. in their study observed 100% sensitivity and accuracy in the differentiation of uric acid from non-uric acid stones, whereas for the differentiation of oxalate from non-oxalate stones, the sensitivity and specificity values were 97.8% and 92.3%, respectively [9]. Hydroxyapatite stones sometimes accompany infections, which could have played a role in affecting the attenuating values substantially [18]. The high accuracy of DECT in the detection of cysteine and uric acid stones has also been endorsed by Erdogan et al. in their study [15]. In the present study, there was no difficulty in diagnosing the mixed types, whereas Manglaviti et al. in their study found difficulty in the correct diagnosis of mixed types in four out of five mixed-type cases, probably owing to the fact that the stone size in their study had a diameter less than 1 cm. In the present study, the average diameter of the stones was close to 1 cm (9.45 mm) and those with mixed type either had multiple stones or had a diameter greater than 1 cm, hence there was no such difficulty in their correct identification [16].

DECT has several important limitations in the characterization of urinary calculi. Accuracy in calculi characterization decreases when evaluating calculi <3-5 mm because it is difficult to obtain accurate attenuation values [19].

Certain secondary signs aid in the diagnosis of ureteral stones on CT, including reliable signs, such as perinephric fat stranding, periureteral edema, hydroureter, and hydronephrosis, and less-consistent signs such as perinephric edema and lateral conal fascial thickening. The PPV and NPV of intra-renal collecting system dilatation and perinephric fat stranding in detecting ureterolithiasis near 98% and 91%, respectively [8].

Such subjective additional signs were taken into account in the index study and thus based on CT images, perinephric edema was subjectively categorized into extensive and limited, which were suggestive of high-grade and low-grade obstruction, respectively. The study results showed that a high-grade obstruction was present in 42% cases while a low-grade obstruction was seen in 58% cases.

It was observed in the present study that agreement between the volume of calculus by volume fluid displacement and DECT assessments showed sensitivity 92.8%, specificity 98.2%, PPV 97.5 %, NPV 95%, and accuracy 96%. On the assessment of the volume of calculi, sensitivity was 92.8% and specificity was 98.2%. The diagnostic accuracy was 96%.

The index study findings are consistent with the study of Boridy IC et al., who reported that limited perinephric edema on CT images had a sensitivity of 88%, a specificity of 100%, PPV of 100%, and NPV of 88% for the prediction that calculus was associated with a low-grade ureteral obstruction, whereas for high-grade ureteral obstruction, CT images had sensitivity 100%, specificity 91%, PPV 83%, and NPV 100% [12]. Similar findings were seen in the studies by Alice E et al. and Leng S et al. [13-14].

It must be kept in mind that the management strategy of the stones is greatly dependent upon the chemical composition of the stones. For instance, uric acid calculi can dissolve in the urine of higher pH and thus alkalization of the urine plays an effective role. On the other hand, another non-invasive management strategy, i.e., extracorporeal shock wave lithotripsy is less effective in the management of oxalic acid stones [20]. Thus, prior knowledge of the volume, degree of obstruction, and chemical composition of the stones can help in determining the route of management and could thus restrict the invasive procedures to the minimum level. The present study showed that DECT could prove to be a useful tool in the morphological, anatomical, as well as chemical characterization of urinary calculi and thus could be a useful tool in guiding the management strategy. It is a common perception that DECT scanning leads to increased ionizing radiation exposure as compared with conventional CT, which limits the wide implementation of DECT. However, it has been seen that radiation dose considerations with DECT are comparable to those achieved with conventional CT. Indeed, DECT exams for urolithiasis can be performed under 5 mSv in ssDECT and dsDECT scanners.

One of the limitations of DECT is that it does not determine the functional status of the renal system, which is an important parameter for the management of ureteric stones. Secondly, the standard DE ratio cutoffs have not been established to characterize the ureteric stones that may affect the reported incidence of the chemical nature of the stones. In addition, we could not incorporate the intraoperative observations regarding the degree of obstruction to validate the overdetection of high grades of obstruction on DECT as compared to a urogram.

## Conclusions

It can be concluded that DECT is a novel non-invasive imaging technique that provides comprehensive information and accurate prediction regarding the morphological, chemical, and anatomical characterization of ureteric stones. In addition, it showed high sensitivity and specificity for determining high-grade obstruction. The findings prompt the use of DECT as a potential tool for advanced treatment planning for urinary obstruction, thereby reducing the burden of surgical intervention.
